# Theoretical research on reasonable shield support capacity in close-multiple coal seams with the coordinated mining: A case study of Qianjiaying coal mine

**DOI:** 10.1371/journal.pone.0276101

**Published:** 2022-10-18

**Authors:** Yang Li, Yuqi Ren, Xinghai Lei, Nan Wang, Xiangyang Jin, Guoshuai Li

**Affiliations:** School of Energy and Mining Engineering, China University of Mining and Technology-Beijing, Beijing, China; University of Vigo, SPAIN

## Abstract

In order to assess the rationality of the rated shield support capacity (RSSC) experienced selection and guide the reasonable RSSC selection for the subsequent working faces of each coal seam, the coupling relationship between shield and roof strata was revealed during each coal seams mining. According to whether the fractured rock blocks generated by the main roof are articulated and whether the upper coal seam has been mined and influenced on the lower coal seam, two roof structure mechanical models of the rock blocks generated by the thick main roof and two calculation methods of a given load on the rock blocks are proposed. In addition, a selection method of roof structure model for maximum shield support capacity (MSSC) of close-multiple coal seams with the coordinated mining is put forward. Three roof structures to calculate the MSSC are established. Based on a case study of close-multiple coal seams with the coordinated mining in the Qianjiaying coal mine, the MSSC is calculated and analyzed in each coal seam combined with roof structure characteristics description, theoretical analysis, and field measurement. No.7, No.12-1, and No.5 coal seams mining are applicable to a voussoir beam balanced structure. No.8 coal seam mining is applicable to a balanced structure with a given load of loose body. No.9 coal seam mining is applicable to a voussoir beam balanced structure with a given load of loose body. Through the calculation, the MSSC of No.7, No.8, No.12-1, No.9, and No.5 coal seam is 3948.55kN, 4018.32kN, 4101.63kN, 3560.03kN, and 4015.30kN, respectively. And the RSSC suggested selection of each coal seam is 4500kN, 4300kN, 4300kN, 4000kN, and 4300kN, respectively. By field measurement, the RSSC experienced selection of each coal seam in the Qianjiaying coal mine is unreasonable with low support load utilization. However, after adopting the RSSC suggested selection in each coal seam, the support load utilization increased by 29.07%, 9.6%, 8.57%, 15.33%, and 11.39%.

## Introduction

Longwall mining for coal seam has grown in popularity in recent years, both at home and abroad, due to its high efficiency and convenience in coal production [1–4]. The hydraulic shield is an important piece of equipment for safe and effective longwall mining [5–11]. The rated shield support capacity (RSSC) is a significant index for longwall mining. When the RSSC is set too high, the cost per ton of coal increases on the one hand, while the shield support design must meet higher specifications on the other hand. When the RSSC is set too low, the stress on the shield exceeds the RSSC, resulting in shield crushing accidents during the weighting of coal seam mining. This will have a serious effect on the safety production of the working face and the lives of miners. As a result, reasonable shield support capacity (RSSC) is crucial to the safety, efficiency, and economics of the working face, mining area, and even the whole mine.

In order to determine the RSSC, it is necessary to determine the maximum shield support capacity (MSSC), also called the shield support capacity (SSC) during the weighting of coal seam mining. The existing determination methods of SSC include the empirical-estimation method, on-site measurement method, voussoir beam structure analysis method, and so on. When using the empirical-estimation determination method, the SSC is approximately estimated by the product of 4~8 times mining height and bulk density. When using on-site measurement determination method, based on a large number of on-site measurement data including the roof-to-floor convergence, periodic weighting interval, and so on, the regression analysis and mathematical-statistical analysis of initial, average, and final SSC are established.

The above determination methods of SSC do not take into account the deformation, movement, fracture, and instability of the roof or overlaying strata. Therefore, some scholars began to focus on the effect of roof strata deformation, movement, fracture, and instability on the SSC, as well as the interaction between the shield and the roof strata. For example, in the late 1990s, Chinese scholar Qian et al. [12] regarded the shield and roof strata as an organic whole and analyzed the coupling mechanism of shield and roof strata to guide shield selection. Cao et al. [13] considered that the subsidence of the main roof was influenced by the change of SSC under the premise that the thickness of the immediate roof was less than 6 times the thickness of the coal seam, which illustrated there was a certain relationship between roof strata and SSC. Song [14] developed and improved the theory of practical mine pressure control, based on the practice and production in the past ten years. A dynamic structure model during coal seam mining was established to determine the SSC. Wang et al. [15, 16] established the binary criterion and believed that SSC needed to balance the load applied by roof strata and maintain the stability of the coal wall. In addition, a new dynamic determination method of SSC was proposed based on the main roof weighting using establishing theoretical models and conducting field measurements. Singh et al. [17] using a numerical model approach predicted the SSC under the condition of the progressive caving behavior of strata. Pang et al. [18] analyzed the coupling relationship and control method of strength, stiffness, and stability between shield and roof strata in the working face based on the coal seam mining with the ultra-large-height mining method. And a “two-factors” determination method of SSC was proposed in ultra-large-height mining. Yan et al. [19, 20] put forward a roof structure called “short cantilever beam and articulated beam structure” based on the new concept and discrimination method of the immediate roof and main roof with large-height mining method. The calculation formula of SSC was also given. Feng et al. [21] developed a face-contacted block structure based on block theory, which promoted the basic theory of upward mining. Zhang et al. [22] analyzed the influence of different key stratum positions on SSC during coal seam mining with large-height top-coal caving method. Huang et al. [23, 24] proposed the “step voussoir beam structure” based on the shallow coal seam mining and studied the stress, displacement, and fracture fields in shallow coal seam group mining. Only the determination method of SSC in single minable coal seam mining was considered in the above research, for example, the thick coal seam mining with full-height mining method, large-height mining method and top-coal caving method. It is noteworthy that the roof structure is not influenced by the mining of the adjacent coal seams during the single coal seam mining. In other words, the SSC in single minable coal seam mining was calculated using the traditional theory of voussoir beam, in the view of which the movement of blocks formed by the main roof breaking was often simplified to be caused by the uniformly distributed load on the blocks.

Generally, there is often more than one mineable coal seam within the coal-bearing strata [25–30]. Because the complexity and variability of stratigraphic conditions have modified the formation and the occurrence of coal seams, the close-multiple coal seams are widely distributed around the world, particularly in China. The mining sequence for close-multiple coal seam is divided into three categories: downward mining [31–34], upward mining [35, 36], and coordinated mining [37–42]. As a result, the aforementioned determination methods of MSSC may be suitable for some coal seams in close-multiple coal seams with a large interlayer distance between each other without mining influence, or the first coal seam to be mined, or the coal seam located at top of the coal-bearing strata in coordinated mining. When the interlayer distance between two adjacent coal seams is small, the aforementioned determination methods of MSSC maybe be unsuitable.

Therefore, when the variation of interlayer distance of each coal seam is large, it is necessary to consider the mining influence to the roof strata in calculation methods of MSSC of each coal seam. Based on this, two roof structure mechanical models are established to calculate the MSSC according to whether the fractured rock blocks generated by the main roof are articulated. In addition, there are two calculation methods of a given load on the rock blocks according to whether the upper coal seam is mined. Furthermore, a selection method of roof structure model for MSSC of close-multiple coal seams with the coordinated mining is put forward. And three roof structures calculation model of the MSSC is established. Combined with a case study of close-multiple coal seams with the coordinated mining in the Qianjiaying coal mine, the MSSC during each coal seam mining is calculated and analyzed. Through field measurement to analyze maximum support load utilization of each coal seam, the rationality of RSSC experienced selection is verified. And the RSSC suggested selection by the theoretical analysis is given. It is effective to guide the RSSC suggestion for the subsequent working faces of each coal seam in the Qianjiaying coal mine. At the same time, the research results may provide a basis for the RSSC suggestion in other close-multiple coal seams mining.

## Classification of roof structure characteristics

The coupling relationship between shield and roof strata is the foundation of ground control in the stope, as well as the basis to verify the effect of roof stability and shield suggestion. Each coal seam mining may be influenced by the adjacent coal seams mining during the close-multiply coal seams with the coordinated mining. However, with the coordinated mining, the lower coal seam mining may be influenced by the upper coal seam mining. When the distance between upper and lower coal seams is large, the integrity of the main roof of the lower coal seam will not be influenced by the upper coal seam mining. The rock blocks generated by the main roof are articulated to form a masonry beam structure during mining. When the distance between upper and lower coal seams is small, the integrity of the main roof of the lower coal seam will be influenced by the upper coal seam mining with many cracks. The rock blocks generated by the main roof are not articulated to form a masonry beam structure during mining. In summary, according to whether the fractured rock blocks generated by the main roof are articulated, there are two types of mechanical models to calculate the MSSC. The first type is the articulated structure of the fractured rock blocks generated by the main roof because the lower coal seam is not influenced by upper coal seam mining. The second type is the unarticulated structure of the fractured rock blocks generated by the main roof because the lower coal seam is influenced by upper coal seam mining.

### Articulated Structure of the Fractured Rock Blocks (ASFRB)

**Fig 1:**
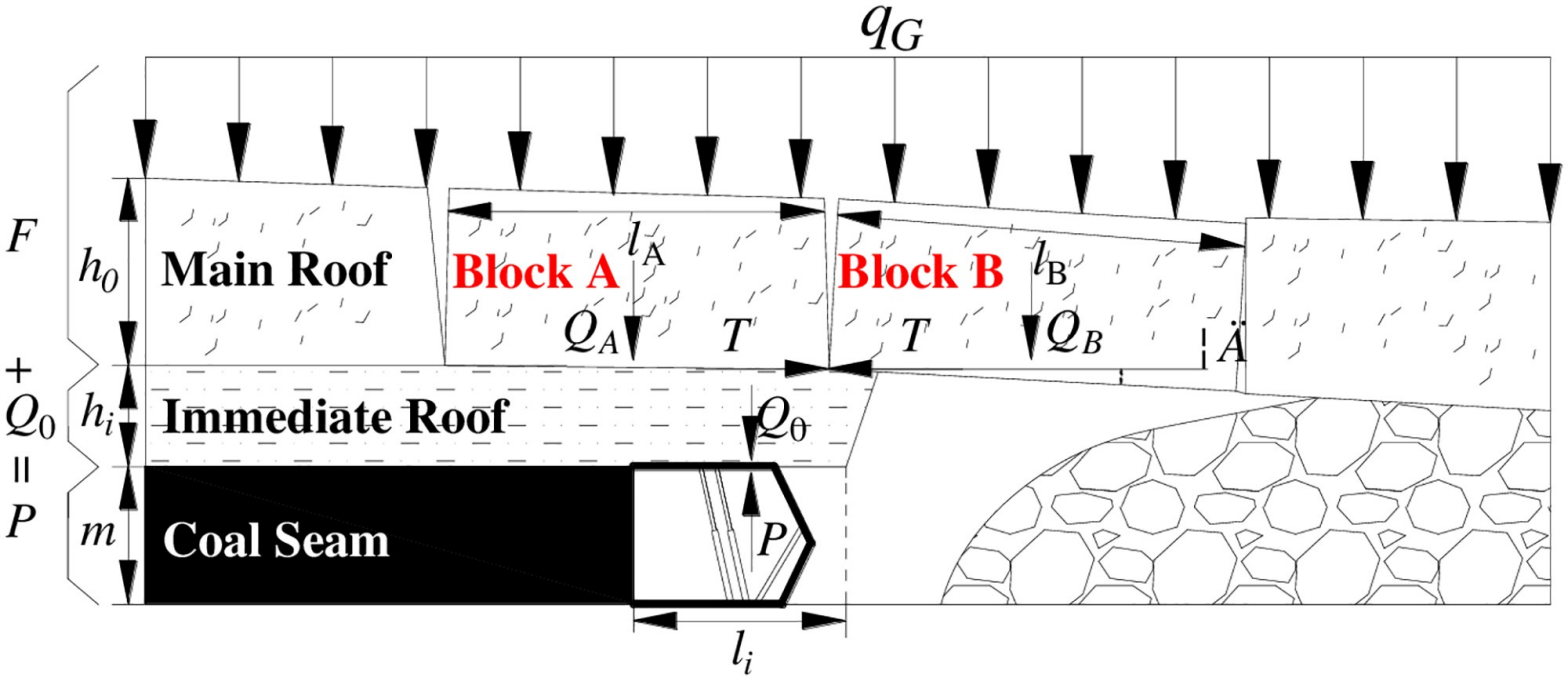
Articulated Structure of the Fractured Rock Blocks (ASFRB).

Fig 1 shows the roof structure characteristic of the ASFRB. When main roof of lower coal seam is not influenced by the upper coal seam mining, the shield-roof strata system is made up of the shield, cantilever beam of the immediate roof, and voussoir beam of the main roof.

That means the MSSC (*P*) per unit width is composed of the load of the immediate roof (*Q*_0_) and the force acting on the shield when the rock blocks of the voussoir beam are generated by thick main roof slip instability (*F*) [43] as Eq (1).

$$P=Q_{0}+F$$ (1)

Where:

*P* is the value of MSSC, kN;

*Q*_*0*_ is the load of the immediate roof, kN;

*F* is the force acting on the shield when the rock blocks of the voussoir beam are generated by thick main roof slip instability, kN.

1. **weight of immediate roof *Q***_***0***_
  *Q*_0_ can be calculated as follows in Eq (2).
$$Q_{0}=\sum_{0}^{i}\gamma_{i}\times h_{i}\times l_{i}$$ (2)
  Where,
  *γ*_*i*_ is the bulk density of *i*^th^ immediate roof, kN /m^3^;
  *h*_*i*_ is the thickness of *i*^th^ immediate roof, m;
  *l*_*i*_ is the length of the *i*^th^ immediate roof, m, which can be calculated as Eq (3).
$$l_{i}=l_{k\text{max}}\times K_{f}$$ (3)
  Where,
  *l*_*kmax*_ is the maximum control distance of shield, m;
  *K*_*f*_ is the allowance coefficient.
  Sometimes, there is no immediate roof under the geological conditions of some coal seams.
2. **force acting on the shield when the rock blocks of the voussoir beam are generated by thick main roof slip instability (*F*)**
  *F* can be calculated as Eq (4).
$$\left\{\begin{array}{l} F=Q_{A+B}-\frac{l_{B}\cdot Q_{B}}{2\left(h_{B}-\delta \right)}\text{tan}\left(\varphi -\theta \right)\\ Q_{A+B}=Q_{A}+Q_{B}+q=\gamma_{A}\times h_{A}\times l_{A}+\gamma_{B}\times h_{B}\times l_{B}+q_{G}\\ \delta =h_{B}+m-K_{p}\sum_{0}^{i}h_{1i}\\ \theta =\text{arctan}\frac{\delta }{l_{B}} \end{array}\right.$$ (4)
  Where:
  *Q*_*A+B*_ is the weight and load of rock blocks A and B, kN;
  *l*_*A*_ and *l*_*B*_ are the length of rock blocks A and B, m;
  *Q*_*A*_ and *Q*_*B*_ are the weight and load of rock blocks A and B, kN;
  *h*_*A*_ and *h*_*B*_ are the thickness of rock blocks A and B, which is also the thickness of the main roof (*h*_*0*_), m;
  *γ*_*A*_ and *γ*_*B*_ are the bulk density of rock blocks A and B, which is also the bulk density of the main roof, kN /m^3^;
  *δ* is the subsidence of rock block B, m;
  *m* is the thickness of the coal seam, m;
  *K*_*p*_ is the bulk factor;
  *φ* is the internal friction angle of the rock blocks A and B, °;
  *θ* is the breaking angle of the rock blocks A and B, °;
  *q*_*G*_ is the load on the rock blocks A and B, kN, which will be further discussed.
  And the *l*_A_ and *l*_B_ can be simplified by using the formula of periodic weighting interval, as Eq (5).
$$l_{A}=l_{B}=l_{0}=h_{0}\sqrt{\frac{R_{T}}{3q_{\text{0}}}}$$ (5)
  Where:
  *l*_*0*_ is the periodic weighting interval of the main roof, m;
  *h*_*0*_ is the thickness of the main roof, m;
  *R*_*T*_ is the tensile strength of the main roof, Pa;
  *q*_*0*_ is the load on the main roof, N/m^2^. Assuming that the width of the main roof is 1 m, the *q*_*0*_ is equal to *q*_*G*_ in Eq (4).

Therefore, when the distance between upper and lower coal seam is large, the rock blocks generated by the main roof are articulated to form a masonry beam structure during mining because the integrity and continuity of the main roof of the lower coal seam is not influenced by the upper coal seam mining.

### Unarticulated Structure of the Fractured Rock Blocks (UASFRB)

**Fig 2:**
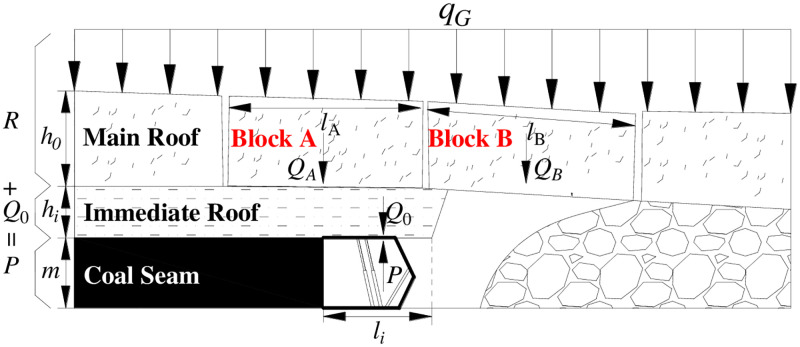
Unarticulated Structure of the Fractured Rock Blocks (UASFRB).


Fig 2 shows the roof structure characteristic of the UASFRB. When the main roof of lower coal seam is influenced by the upper coal seam mining, the shield-roof strata system is made up of the shield, cantilever beam of the immediate roof, and the rock blocks of the main roof. That means the MSSC (*P*) per unit width is also composed of the load of the immediate roof (*Q*_0_) and the force acting on the shield of the rock blocks generated by the thick main roof (*R*) [43] as Eq (6).

$$P=Q_{0}+R$$ (6)

Where:

*P* is the value of MSSC, kN;

*Q*_*0*_ is the load of the immediate roof, kN, which can be calculated as Eq (2).

*R* is the force acting on the shield of the rock blocks generated by the thick main roof, kN.

Similarly, there is no immediate roof under the geological conditions of some coal seams sometimes.

In addition, the force acting on the shield of the rock blocks generated by the thick main roof (*R*) can be calculated as Eq (7).

$$R=Q_{A}+Q_{B}+q=\gamma_{A}\times h_{A}\times l_{A}+\gamma_{B}\times h_{B}\times l_{B}+q_{G}$$ (7)

Where,

*Q*_*A*_ and *Q*_*B*_ are the weight and load of rock blocks A and B, kN;

*γ*_*A*_ and *γ*_*B*_ are the bulk density of rock blocks A and B, which is also the bulk density of the main roof, kN /m^3^;

*h*_*A*_ and *h*_*B*_ are the thickness of rock blocks A and B, which is also the thickness of the main roof (*h*_*0*_), m;

*l*_*A*_ and *l*_*B*_ are the length of rock blocks A and B, m, which can be calculated as Eq (5) approximately;

*q*_*G*_ is the load on the rock blocks A and B, kN, which will be further discussed.

Therefore, when the distance between upper and lower coal seams is small, the rock blocks generated by the main roof are unarticulated to form a masonry beam structure during mining because the integrity and continuity of the main roof of the lower coal seam is influenced by the upper coal seam mining.

## Classification of the load on the rock blocks A and B

Depending on whether the upper coal seam is mined, the load on the rock blocks A and B (*q*_*G*_) in Eqs (4) and (7) can be calculated in two ways. When the upper coal seam has not been mined before, the load on the rock blocks A and B is the given load of the integral roof strata named the given load of overburden strata (*q*_0_). Nevertheless, when the upper coal seam has been mined before, the load on the rock blocks A and B is the given load of loose body (*q*_*s*_) of the gangue.

### A given load of overburden strata (*q*_*0*_)

**Fig 3:**
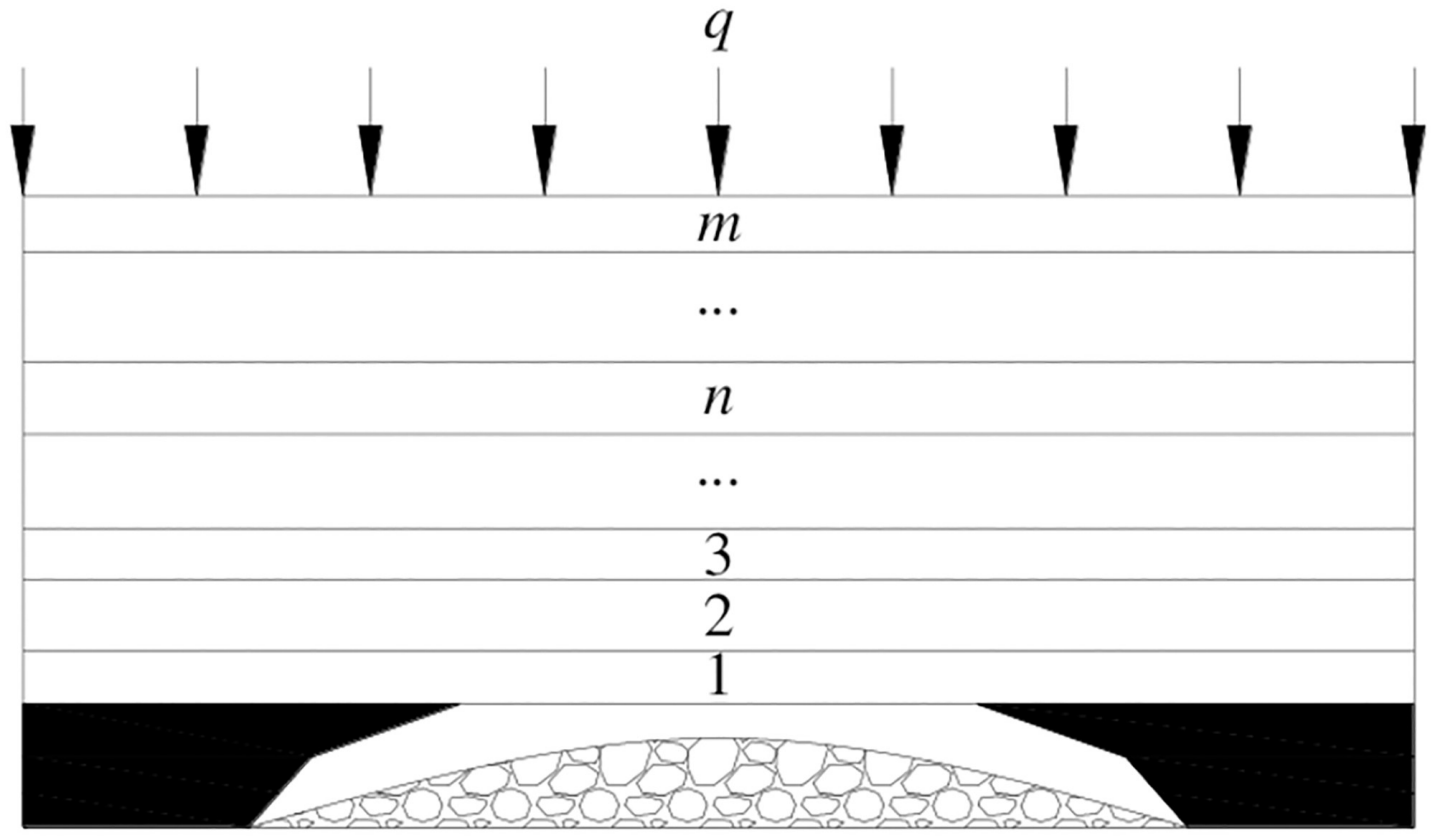
Simplified diagram of the given load of overburden strata.

For the given load of overburden strata, the roof stratum can be transformed into a rock beam for analysis assuming that the load of the stratum is uniformly distributed, as shown in Fig 3.

According to the principle of the composite beam, the shear force (*Q*) and the bending moment (*M*) on each section of the composite beam are borne by the small sections of n rock strata. That is to say.

$$Q=Q_{1}+Q_{2}+\cdots +Q_{n}$$ (8)

$$M_{x}={\left(M_{1}\right)}_{x}+{\left(M_{2}\right)}_{x}+\cdots +{\left(M_{n}\right)}_{x}$$ (9)

Where:

*Q*_*i*_ is the shear force of the *i*^th^ on each section of the composite beam;

*M*_*i*_ is the bending moment of the *i*^th^ on each section of the composite beam.

However, the curvature of each rock stratum under its own weight is different. From the material mechanics theory, the curvature (*k*_*j*_) is,

$$k_{j}=\frac{1}{\rho_{j}}$$ (10)

Where:

*ρ*_*j*_ is the radius of curvature.

And the relationship between curvature and bending moment is,

$$k_{j}=\frac{1}{\rho_{j}}=\frac{{\left(M_{j}\right)}_{x}}{E_{j}J_{j}}$$ (11)

Where:

*E*_*j*_ is the elastic modulus of the *j*^th^ main roof, MPa;

*J*_*j*_ is the section distance of the *j*^th^ neutral axis, m^3^.

When the radius of curvature of each rock stratum is large, the curvature of each rock stratum tends to be consistent due to the combination of rock strata. And the bending moment of each rock stratum is redistributed. That is,

$$\frac{{\left(M_{1}\right)}_{x}}{E_{j}J_{j}}=\frac{M_{1}}{E_{1}J_{1}}=\frac{M_{2}}{E_{2}J_{2}}=\cdots =\frac{M_{n}}{E_{n}J_{n}}$$ (12)

$$\frac{{\left(M_{1}\right)}_{x}}{{\left(M_{2}\right)}_{x}}=\frac{E_{1}J_{1}}{E_{2}J_{2}},\frac{{\left(M_{1}\right)}_{x}}{{\left(M_{3}\right)}_{x}}=\frac{E_{1}J_{1}}{E_{3}J_{3}},\cdots \frac{{\left(M_{1}\right)}_{x}}{{\left(M_{n}\right)}_{x}}=\frac{E_{1}J_{1}}{E_{n}J_{n}}$$ (13)

But,

$$M_{x}={\left(M_{1}\right)}_{x}+{\left(M_{2}\right)}_{x}+\cdots +{\left(M_{n}\right)}_{x}$$ (14)

$$M_{x}={\left(M_{1}\right)}_{x}\left(1+\frac{E_{2}J_{2}+E_{3}J_{3}+\cdots +E_{n}J_{n}}{E_{1}J_{1}}\right)$$ (15)

$${\left(M_{1}\right)}_{x}=\frac{E_{1}J_{1}\cdot M_{x}}{E_{1}J_{1}+E_{2}J_{2}+\cdots +E_{n}J_{n}}$$ (16)

Since $Q=\frac{\text{d}M}{\text{d}x}$ and $q=\frac{\text{d}Q}{\text{d}x}$, so

$${\left(Q_{1}\right)}_{x}=\frac{E_{1}J_{1}\cdot Q_{x}}{E_{1}J_{1}+E_{2}J_{2}+\cdots +E_{n}J_{n}}$$ (17)

$${\left(q_{1}\right)}_{x}=\frac{E_{1}J_{1}\cdot q_{x}}{E_{1}J_{1}+E_{2}J_{2}+\cdots +E_{n}J_{n}}$$ (18)

And the *q*_*x*_ and *J*_*j*_ are calculated as follows.

$$q_{x}=\sum_{j=1}^{n}\gamma_{j}h_{j},j=1,2,\cdots ,n$$ (19)

$$J_{j}=\frac{b_{j}h_{j}{}^{3}}{12},j=1,2,\cdots n$$ (20)

Where:

*γ*_*j*_ is the bulk density of the *j*^th^ stratum, kN /m^3^_;_

*b*_*j*_ is the width of the *j*^th^ stratum, m;

*h*_*j*_ is the thickness of the *j*^th^ stratum, m;

(*q*_*n*_)_*m*_ is the load of stratum *n*^th^ to stratum m^th^. That is,

$$q_{0}={\left(q_{n}\right)}_{m}=\frac{E_{m}h_{m}^{3}(\sum_{j=m}^{n}\gamma_{j}h_{j})}{\sum_{j=m}^{n}E_{j}h_{j}^{3}}$$ (21)

Therefore, when the upper coal seam has not been mined before, the load on the rock blocks A and B may be the given load of the integral roof strata named the given load of overburden strata.

### A given load of loose body (*q*_*s*_)

**Fig 4:**
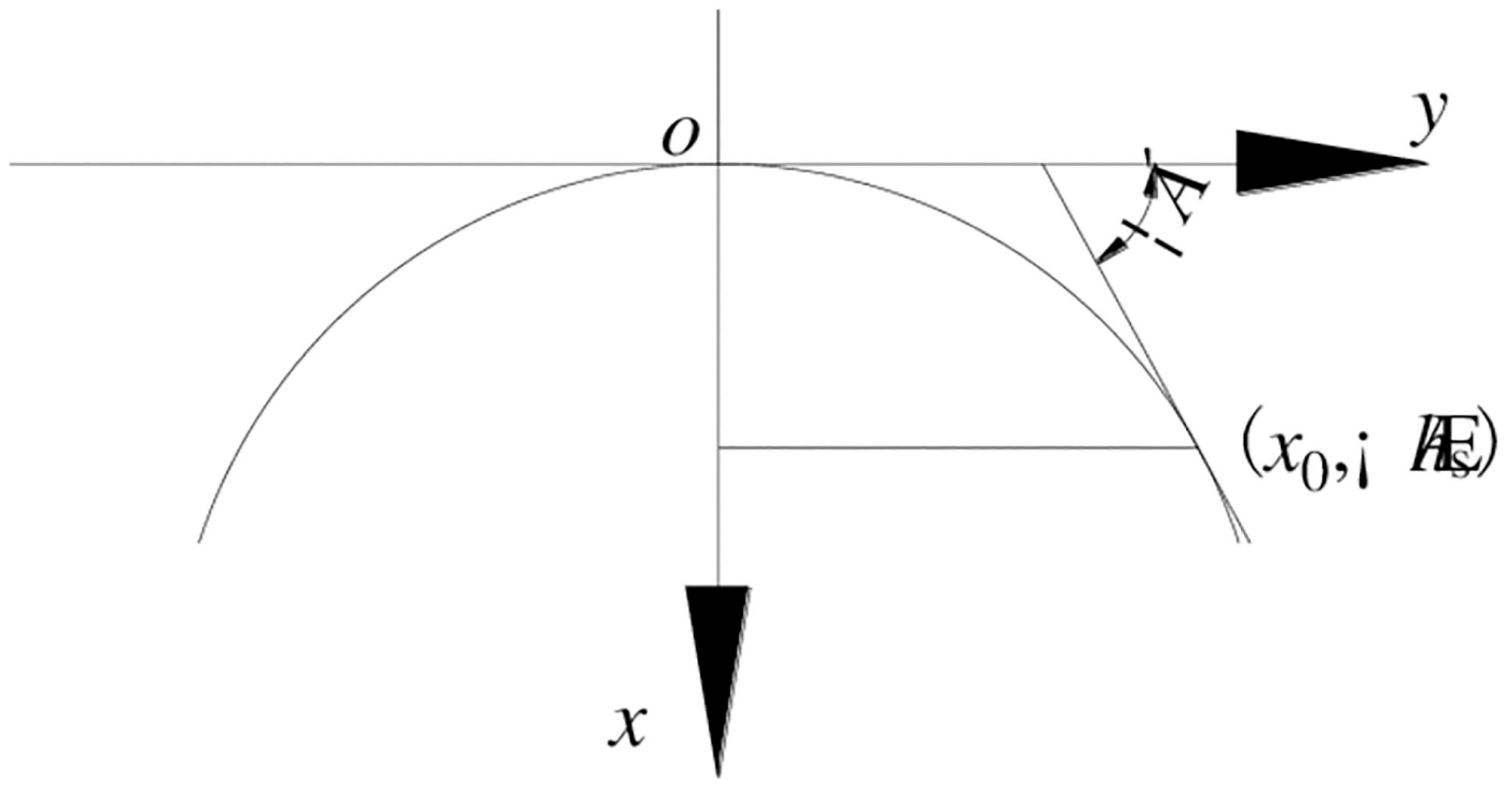
Simplified diagram of the given load of loose body.

For the given load of loose body, the shape of the loose body is idealized to obtain the parabolic shape of bulk arch structure [44, 45], as shown in Fig 4.

It is assumed that the inclination of the tangent at the point (*x*_0_,∑*h*_s_) is approximately the natural repose angle (*α*) of the loose body. At the same time, it is considered that the equation of the parabola is,

$$y=ax^{2}$$ (22)

Bring the point (*x*_0_,∑*h*_s_) to the Eq (22), and the Eq (23) is gotten.


$$\sum h_{s}=ax_{0}^{2}$$ (23)

The derivative of Eq (22) at x = *x*_0_ is:

$$\text{tan}\;\alpha =y\prime \left|{}_{x=x_{0}}=2ax_{0}\right.$$ (24)

So,

$$a=\frac{{\text{tan}}^{2}\alpha }{4\sum h_{s}}$$ (25)

Considering the safety, a correction factor *K*_s_ is added. That is

$$a=K_{s}\frac{{\text{tan}}^{2}\;\alpha }{4\sum h_{s}}$$ (26)

And the parabolic curve equation is

$$y=k_{s}\frac{{\text{tan}}^{2}\;\alpha }{4\sum h_{s}}x^{2}$$ (27)

The area of the parabolic shape of the loose body (*S*) is obtained

$$S=2\left(\sum h_{s}\frac{l_{s}}{2}-\int_{0}^{\frac{l_{s}}{2}}K_{s}\frac{{\text{tan}}^{2}\;\alpha }{4\sum h_{s}}x^{2}dx\right)=\sum h_{s}\cdot l_{s}-\frac{1}{48}K_{s}\frac{{\text{tan}}^{2}\;\alpha }{\sum h_{s}}l_{s}{}^{3}$$ (28)

And the given load in the parabolic shape of the loose body (*q*_s_) is,

$$q_{s}=(\sum h_{s}\cdot l_{s}-\frac{1}{48}K_{s}\frac{{\text{tan}}^{2}\;\alpha }{\sum h_{s}}l_{s}{}^{3})\gamma_{s}$$ (29)

Where,

∑*h*_*s*_ is the height of the loose body, m;

*l*_*s*_ is the span of the loose body, m;

*K*_s_ is the correction factor;

*α* is the natural repose angle of the loose body, °;

*γ*_s_ is the bulk density of the loose body, kN /m^3^.

Therefore, when the upper coal seam has been mined before, the load on the rock blocks A and B may be the given load of loose body of the gangue.

## Selection step of roof structure calculation model of MSSC

**Fig 5:**
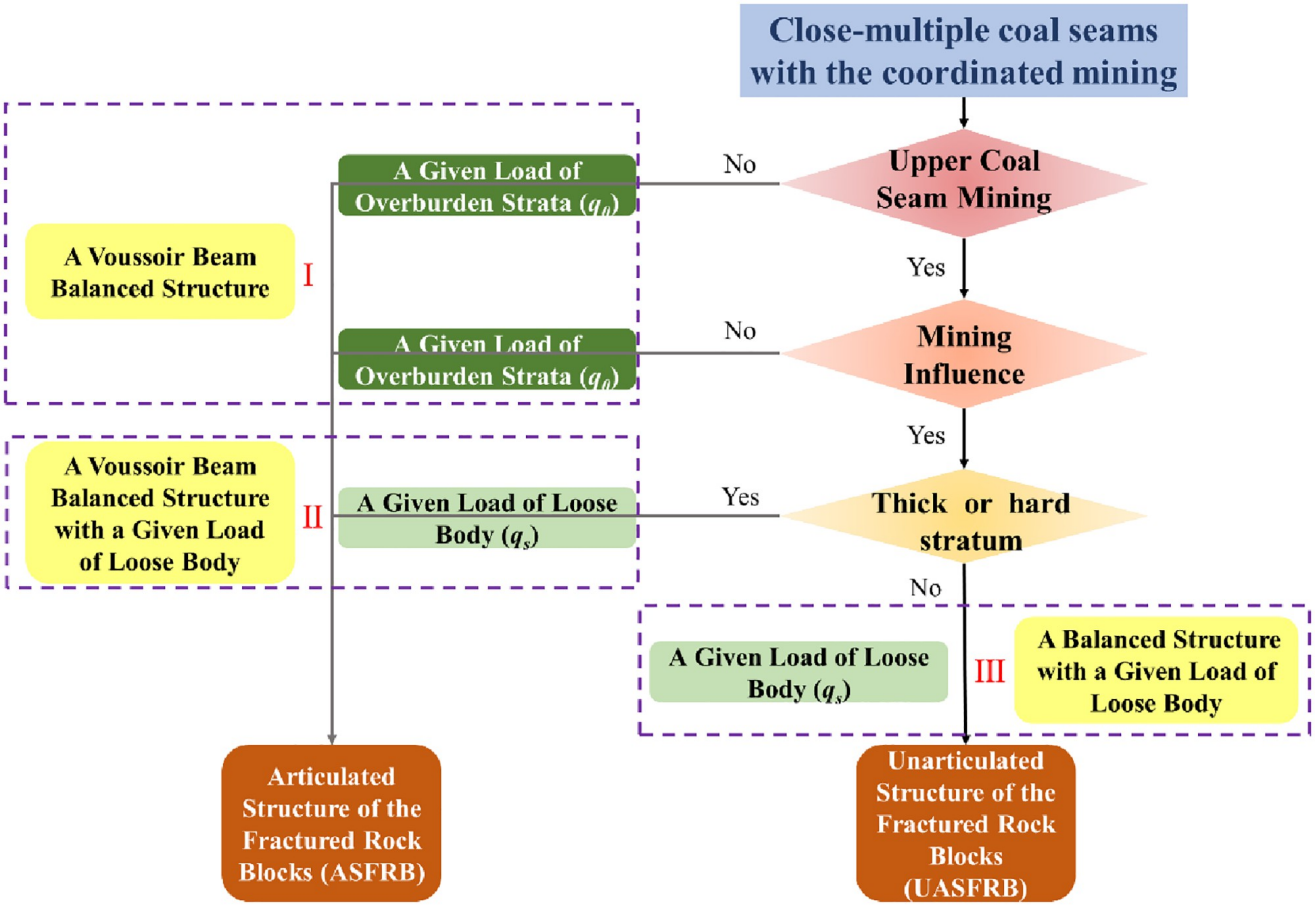
Flow diagram or model selection algorithm.

Fig 5 shows the selection of the calculation method of MSSC. In the close-multiple coal seams mining, there are three main factors that should be considered. In sequence, explore whether the upper coal seam has been mined, study whether the lower coal seam is influenced by the upper coal seam mining. and determine whether there is a thick and hard stratum between the upper coal seam and the lower coal seam. The upper coal seam and the lower coal seam discussed next are adjacent coal seams.

First, if the upper coal seam has not been mined before, the roof structure characteristic is ASFRB during lower coal seam mining, which is always the first coal seam to be mined or the coal seam located at top of the coal-bearing strata. And the load on the rock blocks A and B is a given load of overburden. It is called a voussoir beam balanced structure, as the I shown in Fig 5. On the contrary, if the upper coal seam has been mined before, it is necessary to study whether the lower coal seam is influenced by the upper coal seam mining.

Second, if the lower coal seam is not influenced by the upper coal seam mining, the roof structure characteristic is ASFRB. That also means the interlayer distance between the upper coal seam and the lower coal seam is very large, so the load on the rock blocks A and B is a given load of overburden strata during lower coal seam mining. It is also called a voussoir beam balanced structure, as the I shown in Fig 5. On the contrary, if the lower coal seam is influenced by the upper coal seam mining, the last factor is taken into account.

Depending on whether there is a thick and hard stratum between the upper coal seam and the lower coal seam, the roof structure characteristic is also divided into ASFRB and UASFRB. If there is a thick or hard stratum, the roof structure characteristic is ASFRB during lower coal seam mining. Nevertheless, if there is no thick or hard stratum, the roof structure characteristic is UASFRB during lower coal seam mining. However, because the upper coal seam has been mined and influenced on the lower coal seam mining, the load on the rock blocks A and B is a given load of loose body both in these situations. When the roof structure characteristic is ASFRB and the load on the rock blocks A and B is a given load of loose body, it is called a voussoir beam balanced structure with a given goad of loose body, as the II shown in Fig 5. When the roof structure characteristic is UASFRB and the load on the rock blocks A and B is a given load of loose body, it is called a balanced structure with a given goad of loose body, as the III shown in Fig 5.

According to the selection step to calculate MSSC, there are three roof structures to calculate the MSSC in close-multiple coal seam with the coordinated mining, which are a voussoir beam balanced structure, a balanced structure with a given load of loose body and a voussoir beam balanced structure with a given load of loose body.

## Case study

### Geological and mining condition

**Fig 6:**
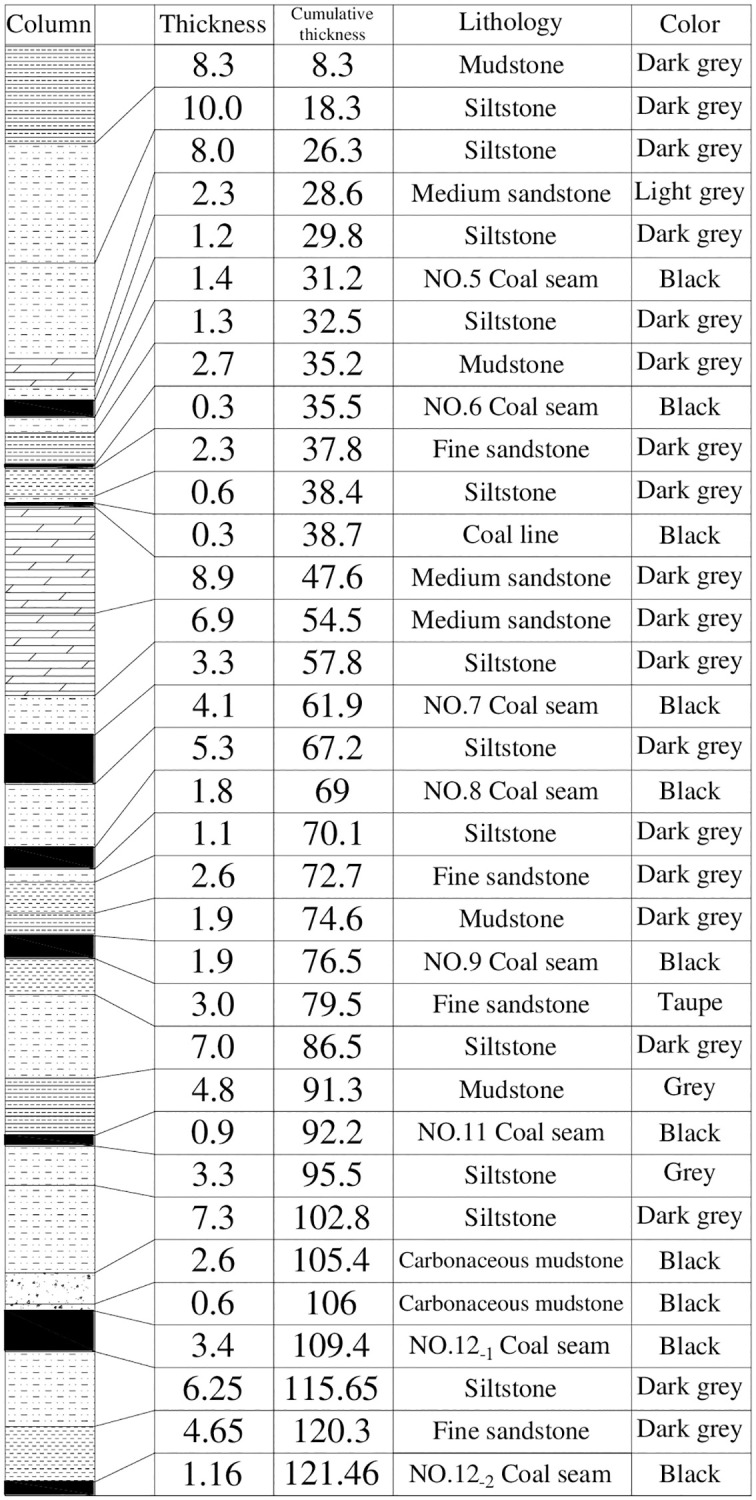
Coal-bearing strata in Qianjiaying coal mine.

Qianjiaying coal mine in the Kailuan coalfield has five mineable coal seams within about 80 m of the coal-bearing strata. From top to bottom, there are No.5 coal seam, No.7 coal seam, No.8 coal seam, No.9 coal seam, and No.12_-1_ coal seam in order. The thickness of each coal seam is 1.4m, 4.1m, 1.8m, 1.9m, and 3.4m, respectively. And the average distance of two adjacent coal seams is about 26.6 m, 5.3m, 5.6m, and 29.5m, respectively. The coal-bearing strata in the Qianjiaying coal mine are shown in Fig 6. The coal seams in the coal-bearing strata are nearly horizontal, so the dip angle of each coal seam is simplified to 0°. Table 1 shows the thickness, quality, and distance of each coal seam. Therefore, typical close-multiple coal seams formed, with characteristics of a small distance between each coal seam and large variations in thickness and quality of each coal seam. To fulfill the dual goal of apportion mining scarce coal and maximizing economic benefits, coordinated mining with downward and upward mining of close-multiple coal seams has been gradually developed in Qianjiaying coal mine on the basis of completely evaluating thickness, quality, distance, production conditions, and economic benefits [40, 41].

**Table 1:** Characterization of occurrence and quality of coal seams.

Coal seam	Average thickness (m)	Ash content	Sulfur content	Calorific value (kCal)	Average distance (m)
No.5	1.4	Low	Low	6000–7000	26.6
No.7	4.1	Low-middle	Low	5300–6200	5.3
No.8	1.8	Middle	Low	5300–6200	5.6
No.9	1.9	Middle	Middle	5500–6500	29.5
No.12_-1_	3.4	Low	Low-middle	5500–6500	

The mining sequence of the close-multiple coal seams in the Qianjiaying coal mine is as follows:

1. No.7 coal seam with large thickness and No.8 coal seam in the upper part of coal-bearing strata were mined. (in some mining areas, No.7 and No.8 coal seams merge)
2. No.12_-1_ coal seam at the bottom was mined.
3. After No.12_-1_ coal seam mining, the No.9 coal seam in the middle part was mined.
4. No.5 coal seam with high-quality at the top was mined.

### Roof structure characteristics of full-cover rock strata

**Fig 7:**
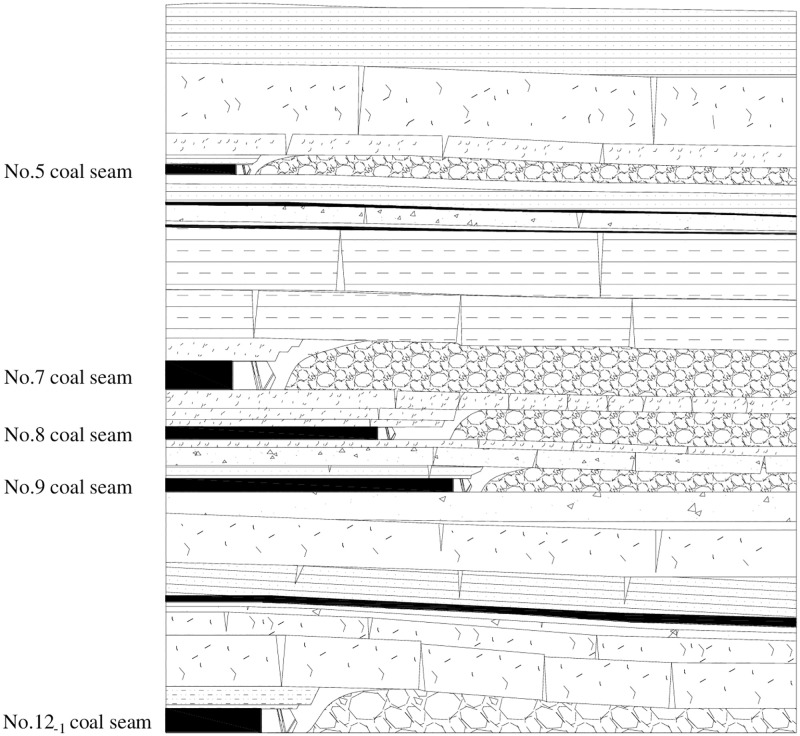
Roof structure model of full-cover rock strata (modified according to [42]).

According to the paper [42] and [46], the roof structure characteristics of full-cover rock strata after coordinated mining of close-multiple coal seams were detected through ground penetrating radar (GPR) and borehole television imaging (BTVI). And a roof structure model of full-cover strata was established, as shown in Fig 7.

From Fig 7, the roof structure characteristics are described after each coal seam mining according to the mining sequence.

#### No.7 coal seam

No.7 coal seam is the first coal seam to be mined in close-multiple coal seams. the immediate roof collapses into gangue as the working face in No.7 coal seam advances. And the thick main roof fractures into blocks when it reaches the limited span. However, the blocks generated by the thick main roof are compressed to each other and subjected to horizontal force and friction due to rotation and reverse rotation. So, the broken blocks are articulated with each other and form a balanced structure.

#### No.8 coal seam

No.8 coal seam is the second coal seam to be mined in close-multiple coal seams with the downward mining. There is just one interlayer stratum of siltstone with a thickness of 5.3 m between the No.8 and No.7 coal seam. Influenced by No.7 coal seam mining, the roof of No.8 coal seam fractures into blocks that cannot be articulated with the advancement of the working face in No.8 coal seam. At the same time, the collapsed gangue in the gob of No.7 coal seam forms a loose body above the roof of No.8 coal seam due to self-weight.

#### No.12_-1_ coal seam

No.12_-1_ coal seam is the third coal seam to be mined in close-multiple coal seams with the downward mining. The distance between No.8 and No.12_-1_ coal seam is about 35.1 m, so the No.12_-1_ coal seam mining may not be influenced by the No.7 and No.8 coal seams mining. That means the roof strata of No.12_-1_ coal seam are complete. Therefore, the roof structure after No.12_-1_ coal seam mining is similar to that after No.7 coal seam mining.

#### No.9 coal seam

No.9 coal seam is the fourth coal seam to be mined in close-multiple coal seams with the upward mining. The roof and floor strata of No.9 coal seam are both influenced by repeated mining of No.7 and No.8 coal seams above it and No.12_-1_ coal seam below it. Therefore, the primary cracks of roof and floor strata are relatively developed without damage to the overall structure. Between the No.9 coal seam and the No.8 coal seam is a thick and hard fine sandstone stratum with a thickness of 2.6 m. So, as the working face in No.9 coal seam advances, the strata beneath the fine sandstone stratum fracture, collapse, and form the gangue in the gob after No.9 coal seam mining, while the strata above the fine sandstone stratum and itself fracture into blocks and forms a balanced structure articulated with each other. Similarly, because the distance between the No.8 and No.9 coal seam is only 5.6 m, the collapsed gangue after No.8 coal seam mining forms a loose body above the balanced structure due to self-weight.

#### No.5 coal seam

No.5 coal seam is the fifth coal seam to be mined in close-multiple coal seams with the upward mining. The No.5 coal seam is located at top of the coal-bearing strata with a distance of 28.9 m between the No.5 and No.7 coal seam. There may be a few cracks in the roof and floor strata influenced by the repeated mining of the other four coal seams. However, the roof and floor strata still maintain a complete state, so the roof structure after No.5 coal seam mining is similar to the No.7 and No.12_-1_ coal seams.

**Fig 8:**
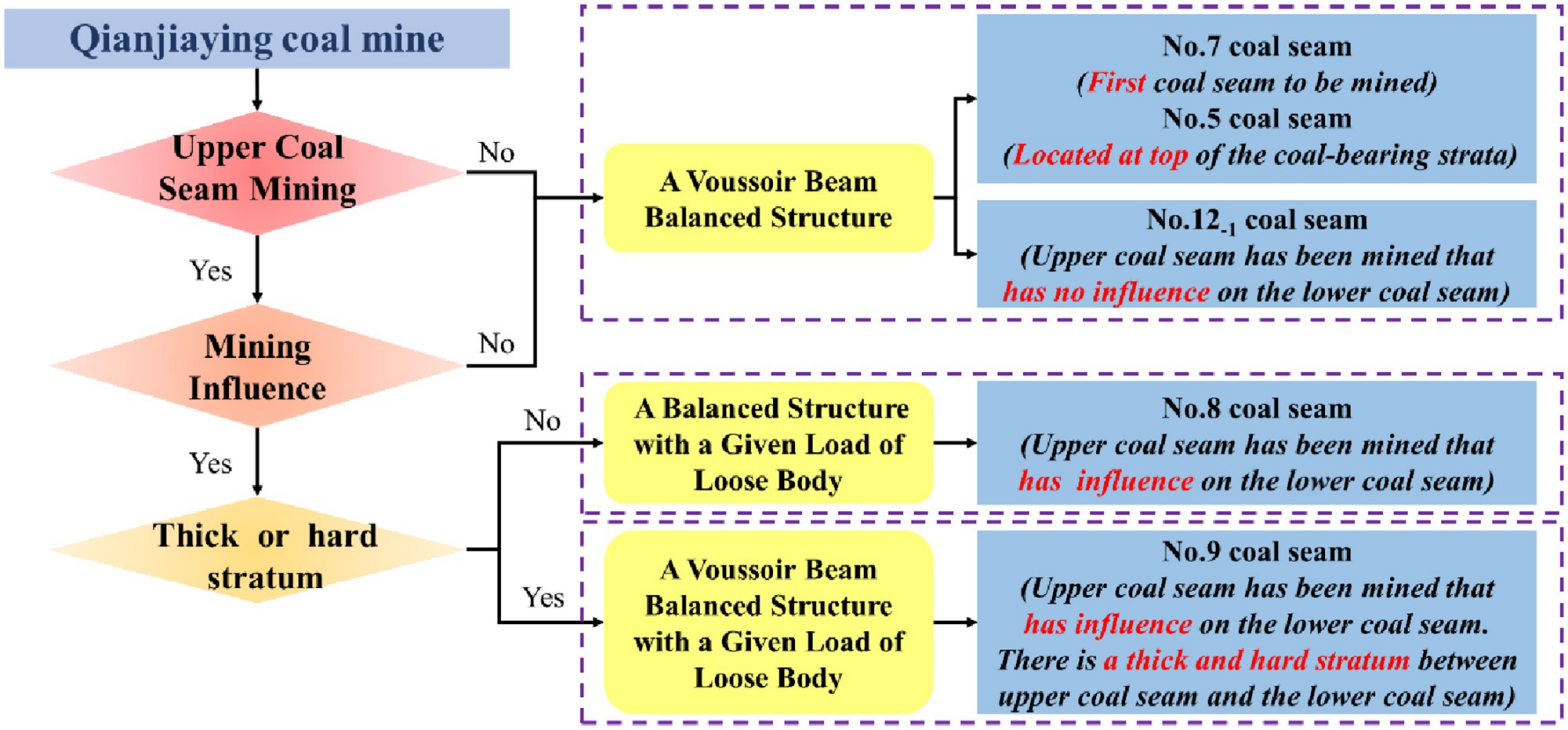
Roof structure calculation model of MSSC in Qianjiaying coal mine.

In summary, taking the close-multiple coal seam with the coordinated mining in Qianjiaying coal mine as a case, the calculation model of MSSC in each coal seam is shown in Fig 8. Through the selection step of roof structure calculation model of MSSC, No.7, No.12-1, and No.5 coal seams mining are applicable to a voussoir beam balanced structure, No.8 coal seam mining is applicable to a balanced structure with a given load of loose body, and No.9 coal seam mining is applicable to a voussoir beam balanced structure with a given load of loose body.

### Analysis of SSC in each coal seam

Considering the width of the shield, the MSSC of each shield is calculated by Eq (30).

$$Q_{M}=Pb$$ (30)

Where,

*Q*_*M*_ is the MMSC considering the width of the shield, kN;

*b* is the width of the shield, m.

And the following analysis in this part is carried out according to the mining sequence of each coal seam.

#### No.7 coal seam

**Fig 9:**
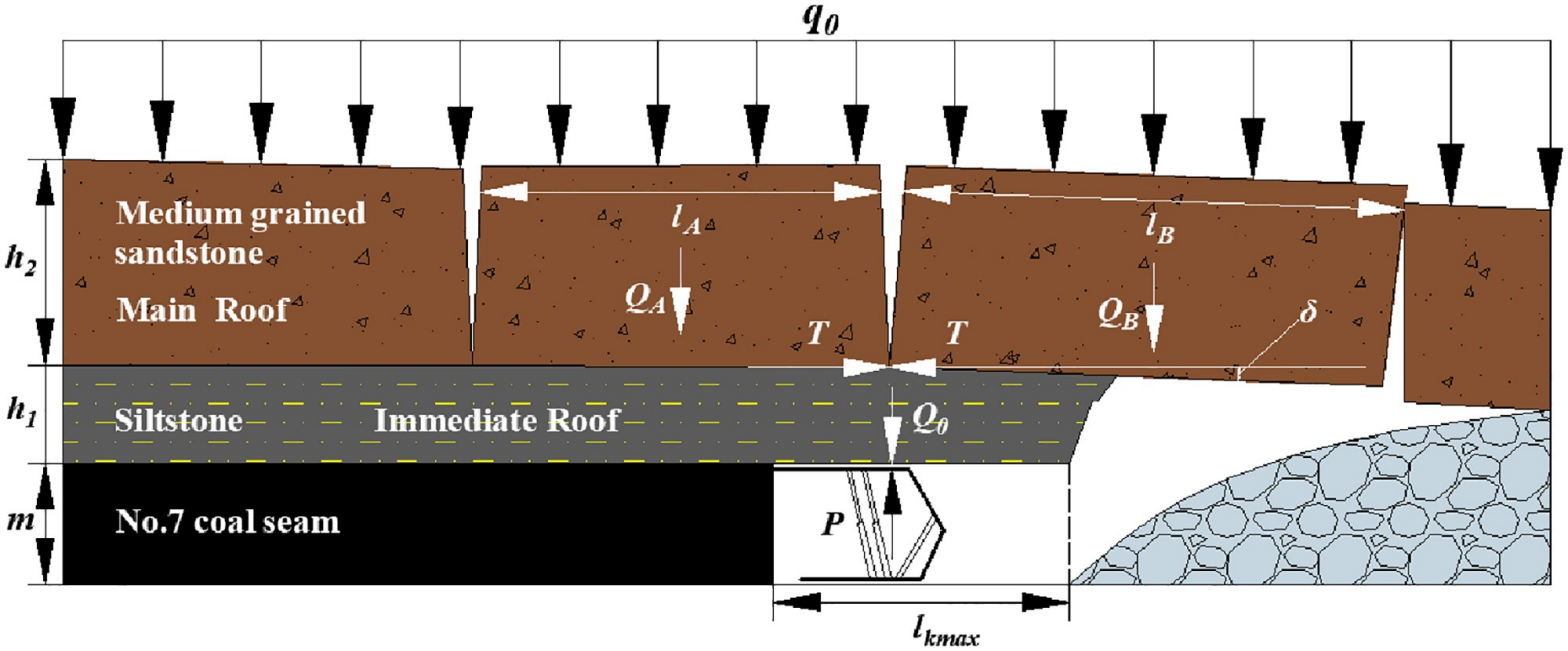
Roof structure characteristic after No.7 coal seam mining.

Fig 9 shows the roof structure characteristic after No.7 coal seam mining. The interaction system between the shield and roof strata is composed of the shield, cantilever beam of the immediate roof, and voussoir beam of the main roof. And the load on the rock blocks A and B of the voussoir beam of the main roof is the given load of overburden strata (*q*_*0*_). The roof structure is named the voussoir beam balanced structure. And the MSSC (*P*) includes the weight of the immediate roof (*Q*_*0*_) and the force acting on the shield when the rock blocks of the voussoir beam are generated by thick main roof slip instability (*F*).

Table 2 shows the relevant parameters and information about the roof strata above No.7 coal seam.

**Table 2:** Physical and mechanical parameters of roof strata above No.7 coal seam.

Number	Name	Lithology	Bulk density *γ*_*i*_ (kN/m^3^)	Thickness *h*_*i*_ (m)	Elastic modulus *E*_*i*_ (kPa)	Tensile strength *R*_*Ti*_ (kPa)
1	Immediate roof	Siltstone	23	3.3	2.1×10^7^	4.2×10^3^
2	Main roof	Medium grained sandstone	24	6.9	2.3×10^7^	5.1×10^3^
3		Medium grained sandstone	25	8.9	2.5×10^7^	5.4×10^3^

According to Eq (21), a load of roof strata on the main roof of the No.7 coal seam can be calculated, as shown in Table 3.

**Table 3:** A load of overburden strata on the main roof of No.7 coal seam.

(*q*_*n*_)_*2*_	*q*_*2*_	(*q*_*3*_)_*2*_
Load of overlying strata on main roof /kPa	165.6	116.46

From Table 3, the load of the third stratum (in Table 2) on the second stratum (main roof, in Table 2) is less than the load of the second stratum itself, hence the third stratum is deemed as a key stratum. That means the third stratum has no load on the second stratum, which is *q*_*0*_ is 0kPa. In addition, the periodical weighting interval of the main roof (*l*_0-7_) of No.7 coal seam is 22.11m calculated by Eq (5). Through Eqs (1), (2), (4), (21), and (30), the MSSC in No.7 coal seam is 3948.55kN based on the calculation parameters in Table 4.

**Table 4:** Calculation parameters of SSC in No.7 coal seam.

*m*	*γ*_1_	*h*_1_	*γ*_2_	*h*_2_	*l*_A_ *= l*_B_ *= l*_0-7_	*φ*	*K*_p_	*l*_kmax_	*K*_f_	*b*
4.1m	23kN/m^3^	3.3m	24kN/m^3^	6.9m	22.11m	33°	1.3	5.1m	1.5	1.5m

#### No.8 coal seam

**Fig 10:**
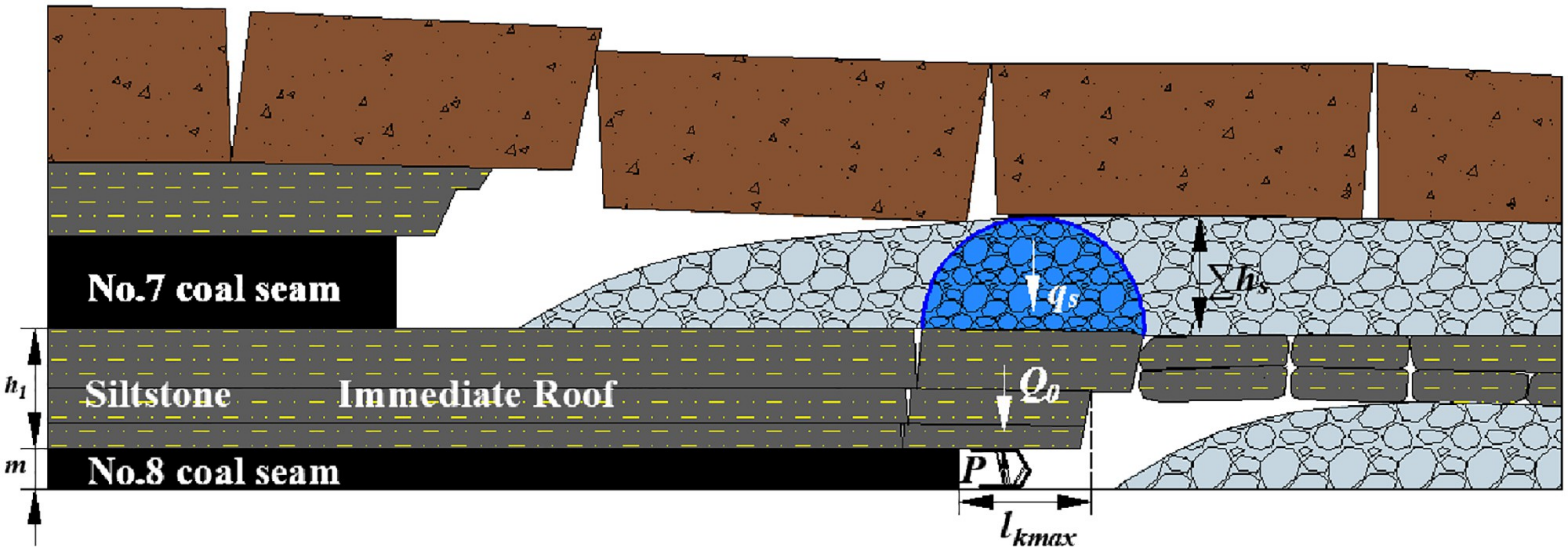
Roof structure characteristic after No.8 coal seam mining.

Fig 10 shows the roof structure characteristic after No.8 coal seam mining. The interaction system between the shield and roof is composed of the shield and a cantilever beam of the roof. There is no main roof above the No.8 coal seam, the given load of loose body (*q*_*s*_) acts directly on the immediate roof. The roof structure is named the balanced structure with a given load of loose body. And the MSSC includes the weight of the immediate roof (*Q*_*0*_) and the force acting on the shield of the rock blocks generated by thick main roof (*R*).

Table 5 shows the relevant parameters and information about the roof strata above No.8 coal seam.

**Table 5:** Physical and mechanical parameters of roof strata above No.8 coal seam.

Number	Lithology	Bulk density *γ*_i_ (kN/m^3^)	Thickness *h*_i_ (m)	Tensile strength *R*_Ti_ (kPa)
1	Siltstone	23	5.3	2.1×10^3^

Because the roof stratum of No.8 coal seam is thick and hard enough, the span of loose body structure (*l*_*s*_) above the roof stratum can be simplified as the limit span of the main roof and calculated by Eq (5). The load of the roof itself is 121.9 kPa calculated with Eq (21). So, the limit span of the roof of No.8 coal seam is 12.7m. Through Eqs (2), (6), (7) (29) and (30), the MSSC in No.8 coal seam is 4018.32kN based on the parameters in Table 6.

**Table 6:** Calculation parameters of MSSC in No.8 coal seam.

*m*	*γ*_1_	*h*_1_	*α*	*γ*_s_	*l*_s_	*K*_s_	∑*h*_*s*_	*b*
1.8m	25kN/m^3^	5.3m	20°	21kN/m^3^	12.7m	1.1	4.95m	1.5m

#### No.12_-1_ coal seam

**Fig 11:**
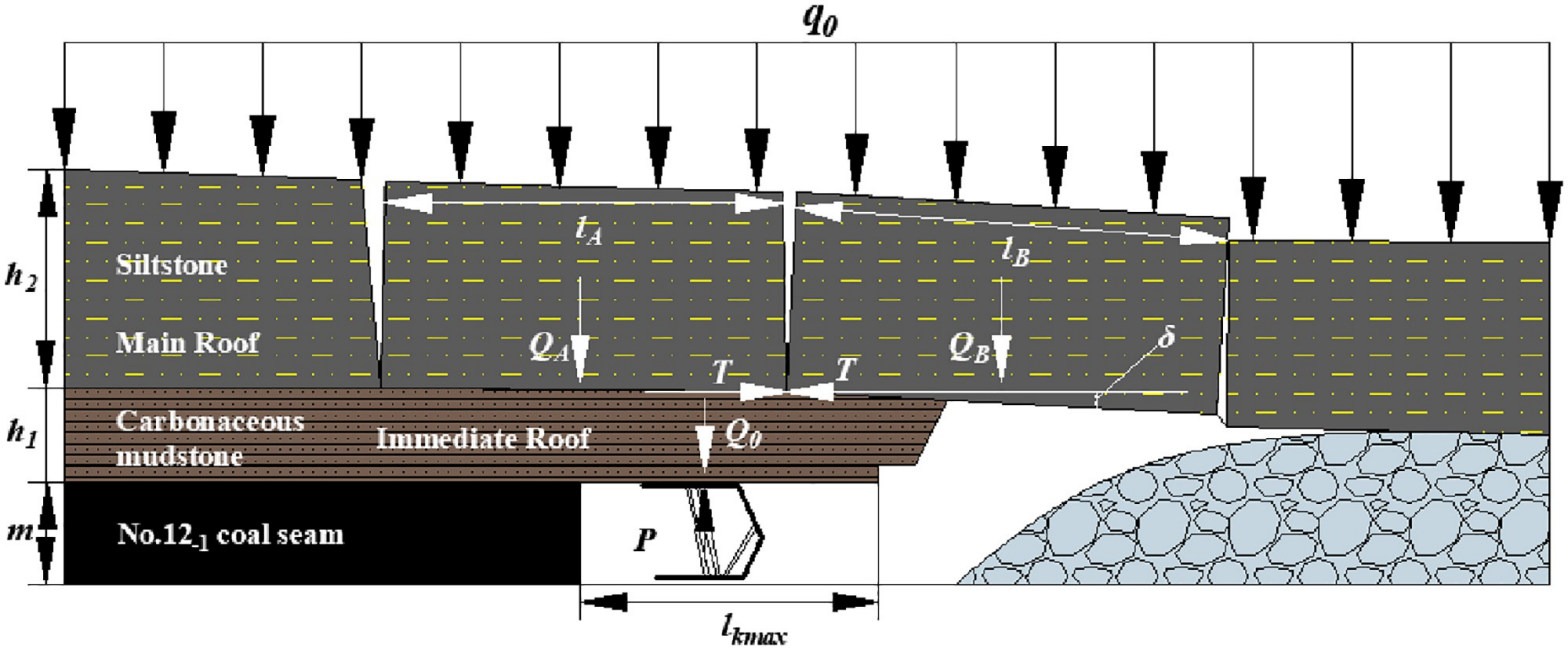
Roof structure characteristic after No.12_-1_ coal seam mining.

Fig 11 shows the roof structure characteristic after No.12_-1_ coal seam mining. The interaction system between the shield and roof strata is composed of the shield, cantilever beam of the immediate roof, and voussoir beam of the main roof. And the load on the rock blocks A and B of the voussoir beam of the main roof is the given load of overburden strata (*q*_*0*_). The roof structure is named the voussoir beam balanced structure. And the MSSC (*P*) includes the weight of the immediate roof (*Q*_*0*_) and the force acting on the shield when the rock blocks of the voussoir beam are generated by thick main roof slip instability (*F*).

Table 7 shows the relevant parameters and information about the roof strata above No.12_-1_ coal seam.

**Table 7:** Physical and mechanical parameters of roof strata above No.12_-1_ coal seam.

Number	Name	Lithology	Bulk density *γ*_i_ (kN/m^3^)	Thickness *h*_i_ (m)	Elastic modulus *E*_i_ (kPa)	Tensile strength *R*_Ti_ (kPa)
1	False roof	Carbonaceous mudstone	23	0.6	1.5×10^7^	2×10^3^
2	Immediate roof	Carbonaceous mudstone	23	2.6	1.5×10^7^	2×10^3^
3	Main roof	Siltstone	25	7.3	2.5×10^7^	3.5×10^3^
4		Siltstone	25	3.3	2.5×10^7^	3.5×10^3^
5		No.11 coal seam	14	0.9	0.5×10^7^	0.03×10^3^
**6**		**mudstone**	**26**	**4.8**	**1.1×10** ^**7**^	**1.8×10** ^**3**^
7		siltstone	25	7.0	2.5×10^7^	3.5×10^3^
8		Fine sandstone	22	3.0	2.4×10^7^	4×10^3^

According to Eq (19), a load of overburden strata on the main roof of No.12_-1_ coal seam can be calculated, as shown in Table 8.

**Table 8:** A load of overburden strata on the main roof of No.12_-1_ coal seam.

(*q*_n_)_3_	*q*_3_	(*q*_4_)_3_	(*q*_5_)_3_	(*q*_6_)_3_	(*q*_7_)_3_
Load of overlying strata on the main roof /kPa	182.5	242.6	254	330.4	275

From Table 8, the load of the sixth stratum (in Table 7) on the third stratum (in Table 7) is less than the load of the seventh stratum (in Table 7) on the third stratum (in Table 7), hence the load on the main roof of No.12_-1_ coal seam (*q*_*0*_) is 330.40kPa. In addition, the periodical weighting interval of the main roof of No.12_-1_ coal seam is 13.4m calculated by Eq (5). Through Eqs (1), (2), (4), (21) and (30), the MSSC in No.12_-1_ coal seam is 4101.63kN based on the parameters in Table 9.

**Table 9:** Calculation parameters of MSSC in No.12_-1_ coal seam.

*m*	*γ*_1_	*h*_1_	*γ*_2_	*h*_2_	*l*_A_ *= l*_B_ *= l*_0-12_	*φ*	*K*_p_	*l*_kmax_	*K*_f_	*b*
3.4m	23kN/m^3^	2.6+0.6m	25kN/m^3^	7.3m	13.4m	46°	1.3	5.1m	1.5	1.5m

#### No.9 coal seam

**Fig 12:**
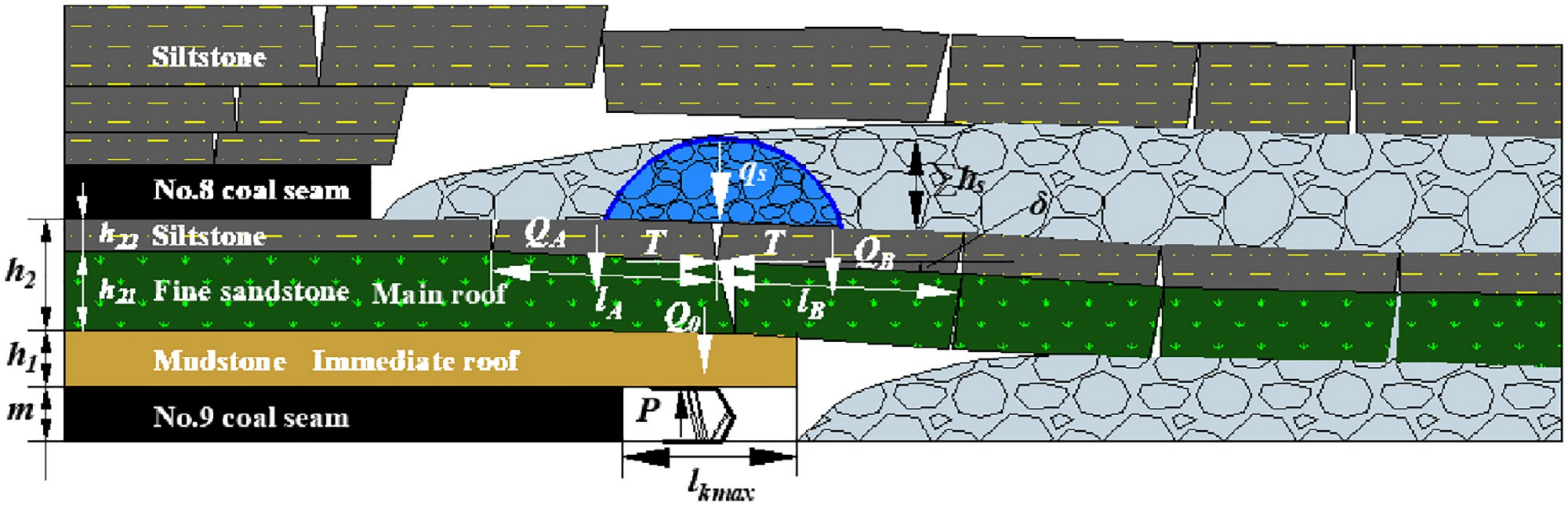
Roof structure characteristic after No.9 coal seam mining.

Fig 12 shows the roof structure characteristic after No.9 coal seam mining. The interaction system between the shield and roof strata is composed of the shield, cantilever beam of the immediate roof, and voussoir beam of the main roof. And the load on the rock blocks A and B of the voussoir beam of the main roof is a given load of loose body of the gangue (*q*_*s*_) in the gob after No.8 coal seam mining. The roof structure is named the voussoir beam balanced structure with a given load of loose body. And the MSSC (*P*) includes the weight of the immediate roof (*Q*_*0*_) and the force acting on the shield when the rock blocks of the voussoir beam are generated by thick main roof slip instability (*F*).

Table 10 shows the relevant parameters and information about the roof strata above the No.9 coal seam.

**Table 10:** Physical and mechanical parameters of roof strata above No.9 coal seam.

Number	Name	Lithology	Bulk density *γ*_i_ (kN/m^3^)	Thickness *h*_i_ (m)	Tensile strength *R*_Ti_ (kPa)
1	Immediate roof	Mudstone	22	1.9	2..5×10^3^
2	Main roof	Fine sandstone	24	2.6	4.8×10^3^
3		Siltstone	24	1.1	5.2×10^3^

The No.9 coal seam is mined after mining the upper No.8 coal seam and lower No.12_-1_ coal seam. Because of the mining influence on the roof and floor strata, the primary cracks in the roof and floor strata are relatively developed without damage to the overall structure. Therefore, the breakage of the second and third stratum in Table 10 are not influenced by each other. Therefore, the load and the breaking length of each stratum can be calculated independently by Eqs (21) and (5). Table 11 shows the calculation results.

**Table 11:** Load and limited span result of each stratum above No.9 coal seam.

Lithology	*q* (kPa)	*l* (m)
Fine sandstone	62.4	13.17
Siltstone	26.4	8.91

So, the breaking length of the main roof is 13.17m. Through Eqs (1), (2), (4), (29) and (30). the MSSC in No.9 coal seam is 3560.03kN based on the parameters in Table 12.

**Table 12:** Calculation parameters of MSSC in No.9 coal seam.

***m***	***γ***_**1**_	***h***_**1**_	***γ***_**21**_	***h***_**21**_	***γ***_**22**_	***h***_**22**_	***l***_**A**_ ***= l***_**B**_ ***= l***_**0-9**_	***φ***
1.9m	21 kN/m^3^	1.9m	24 kN/m^3^	2.6m	24 kN/m^3^	1.1m	13.17m	28
***K***_**p**_	***l***_**kmax**_	***K***_**f**_	***α***	**∑*h***_***s***_	***l***_**s**_ **= *l***_**0-9**_	***K***_**s**_	***γ***_**s**_	***b***
1.3	5.1m	1.5	20°	5.83m	13.17m	1.1	20kN/m^3^	1.5m

#### No.5 coal seam

**Fig 13:**
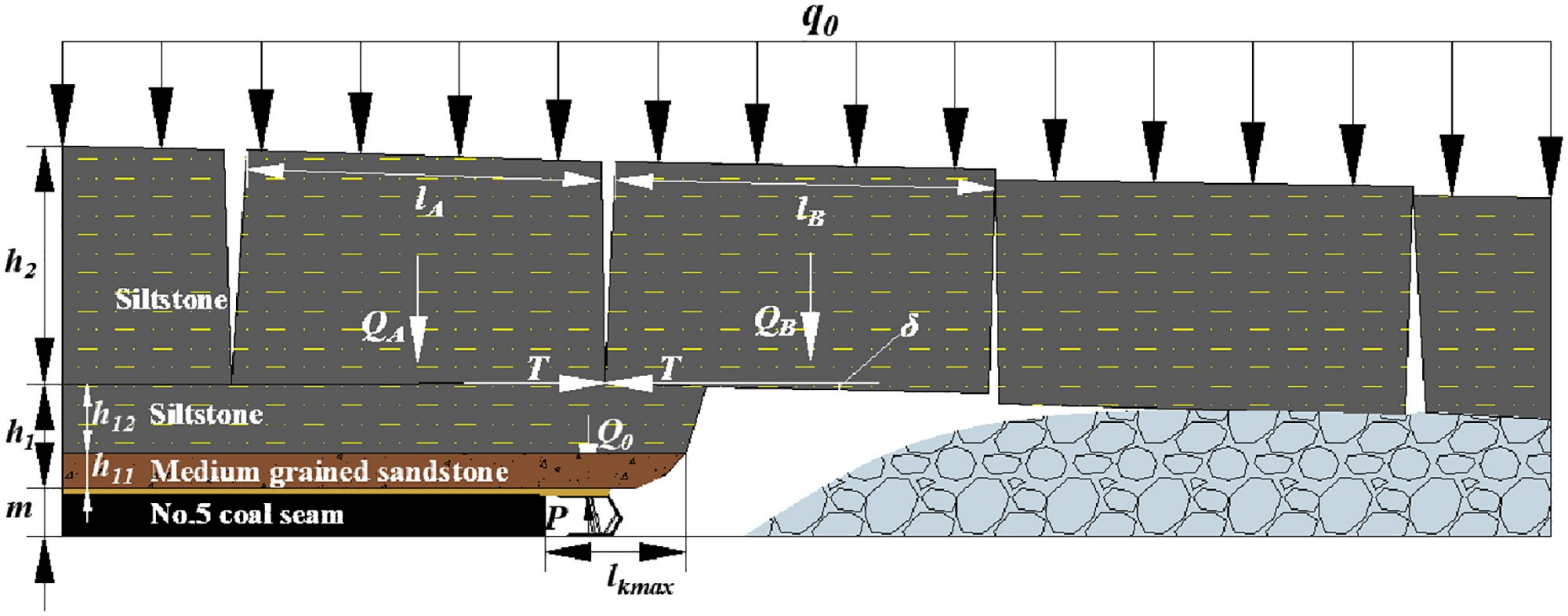
Roof structure characteristic after No.5 coal seam mining.

Fig 13 shows the roof structure characteristic after No.5 coal seam mining. The interaction system between the shield and roof strata is composed of the shield, cantilever beam of the immediate roof, and voussoir beam of the main roof. And the load on the rock blocks A and B of the voussoir beam of the main roof is the given load of overburden strata (*q*_*0*_). The roof structure is named the voussoir beam balanced structure. And the MSSC (*P*) includes the weight of the immediate roof (*Q*_*0*_) and the force acting on the shield when the rock blocks of the voussoir beam are generated by thick main roof slip instability (*F*).

Table 13 shows the relevant parameters and information about the roof strata above No.5 coal seam.

**Table 13:** Physical and mechanical parameters of overburden strata above No.5 coal seam..

Number	Name	Lithology	Bulk density *γ* (kN/m^3^)	Thickness *h* (m)	Elastic modulus *E* (kPa)	Tensile strength *R*_T_ (kPa)
1	Immediate roof	Siltstone	21	1.2	2.3×10^7^	4.6×10^3^
2		Medium grained sandstone	25	2.3	2.35×10^7^	4.4×10^3^
3	Main roof	Siltstone	22	8.0	2.1×10^7^	4.5×10^3^
4		Siltstone	24	10.0	2.4×10^7^	4.8×10^3^

According to Eq (21), a load of overlying strata on the main roof of the No.5 coal seam can be calculated, as shown in Table 14.

**Table 14:** A load of overlying strata on the main roof of No.5 coal seam.

(*q*_n_)_3_	*q*_3_	(*q*_4_)_3_
Load of overlying strata on the main roof /kPa	176	128.71

From Table 14, the load of the fourth stratum (in Table 14) on the third stratum (main roof, in Table 14) is less than the load of the second stratum itself, hence the fourth stratum is deemed as a key stratum. That means the fourth stratum has no load on the third stratum, which is *q*_*0*_ is 0kPa. In addition, the periodical weighting interval of the main roof of No.5 coal seam is 23.35m calculated by Eq (5). Through Eqs (1), (2), (4), (21) and (30), the MSSC in the No.5 coal seam is 4015.3kN based on the calculation parameters in Table 15.

**Table 15:** Calculation parameters of MSSC in No.5 coal seam.

*m*	*γ*_11_	*h*_11_	*γ*_12_	*h*_12_	*γ*_2_	*h*_2_	*l*_A_ *= l*_B_ *= l*_0-12_	*φ*	*K*_p_	*l*_kmax_	*K*_f_	*b*
1.4m	21kN/m^3^	1.2m	25kN/m^3^	2.3m	22kN/m^3^	8m	23.35m	44°	1.3	5.1m	1.5	1.5m

### Field measurement and discussion

#### Measured scheme

**Fig 14:**
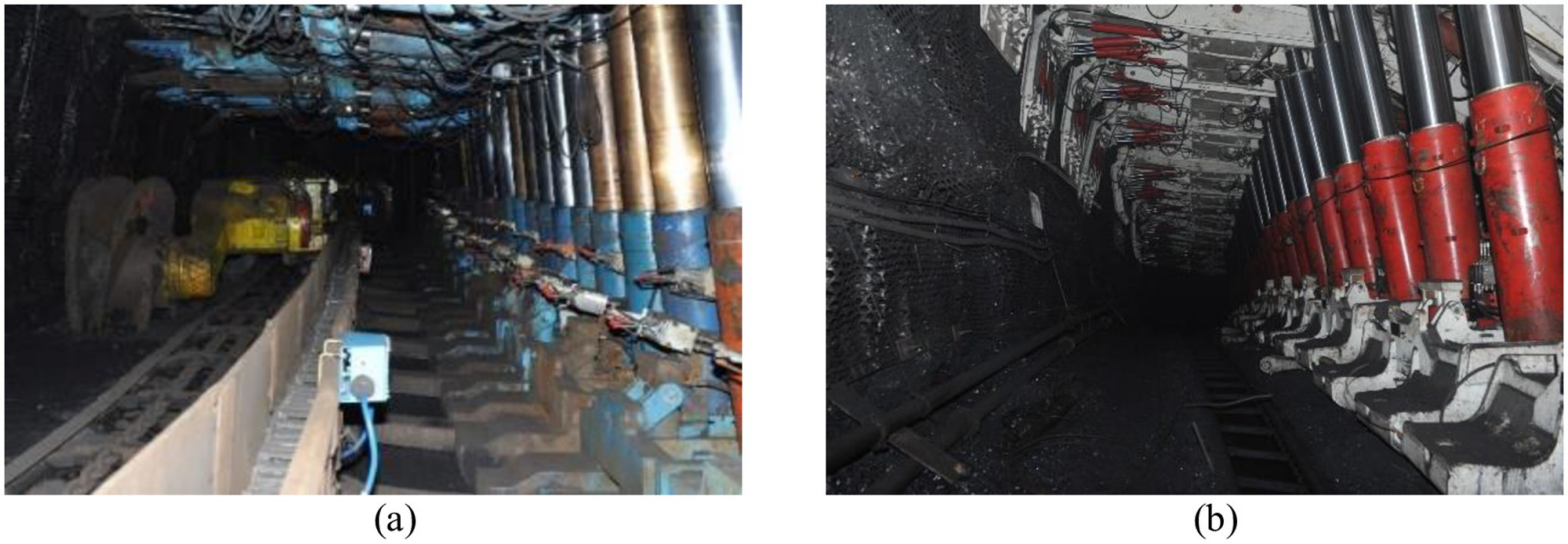
Shield in some typical coal seams. (a) ZY6400-21/45 in No.7 coal seam; and (b) ZY4800-13/32 in No.8 coal seam.

In order to analyze the rationality of the shield selection, the field measurement of the SSC was conducted on the working face of each coal seam. Table 16 shows the length of each working face and the number of measurement stations in each coal seam. The pressure in the left and right columns is measured using the pressure gauges installed on the columns. The actual shield support pressure (ASSP) is calculated by the average value of the left and right column pressures. According to the user handbook of each type of shield in each coal seam, ASSP can be converted to the SSC. From the type of shield in Table 16, the RSSC in each coal seam is 6400kN, 4800kN, 4800kN, 4800kN, and 5000kN, respectively, which is determined by mining experience and called RSSC experienced selection in the following content. Fig 14 shows the shields in some typical coal seams.

**Table 16:** Overview of the working faces in each coal seam.

Coal seam	Working face	Type of shield	Length of working face	Number of stations	Measured distance
No.7	2874	ZY6400-21/45	220m	12	200m
No.8	2882	ZY4800-13/32	180m	10	160m
No.12_-1_	1326	ZY4800-19/40	180m	10	160m
No.9	2196	ZY4800-13/32	200m	13	180m
No.5	1356	ZY5000-09/20D	160m	10	150m

#### Results and discuss

**Fig 15:**
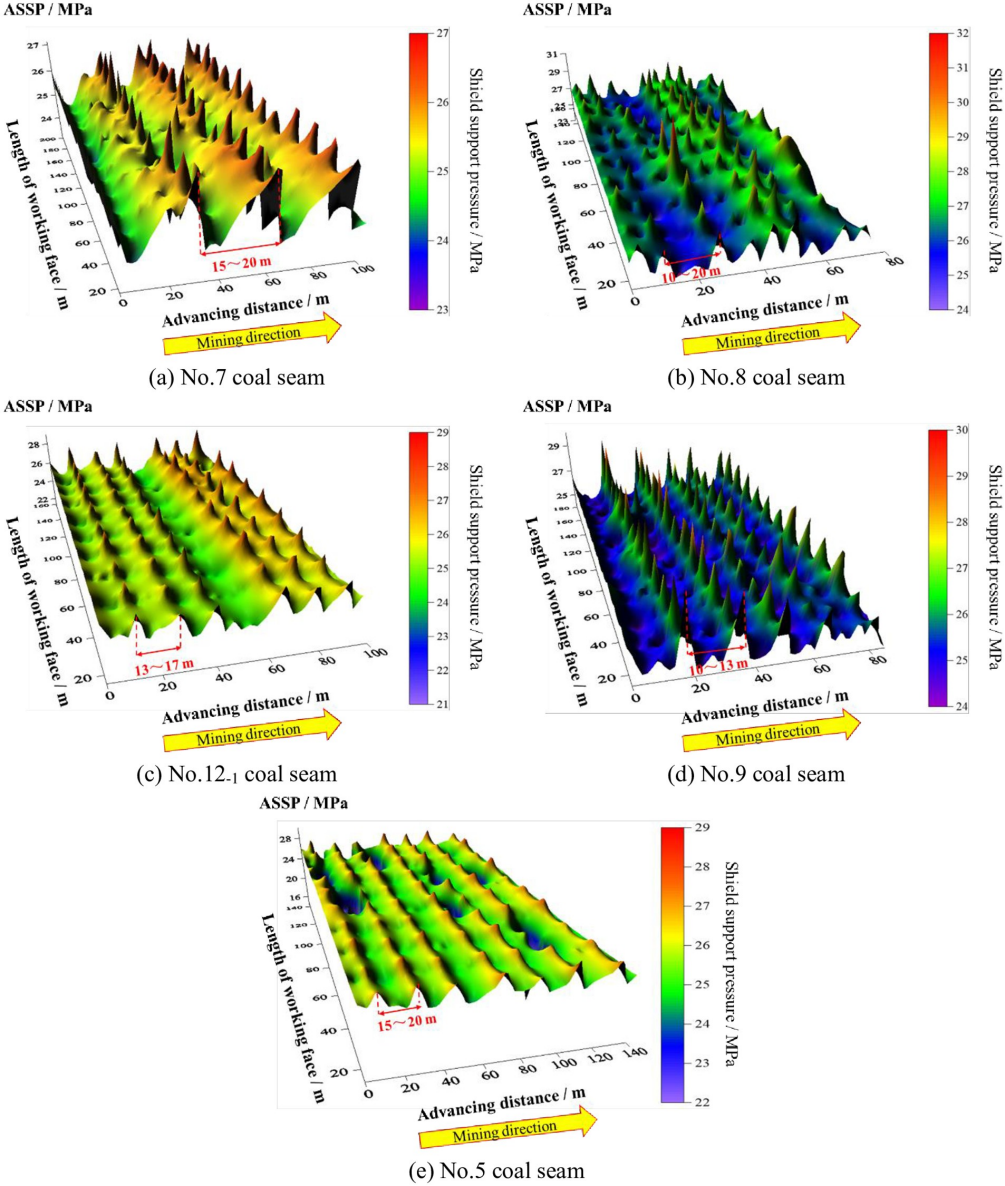
Field measurement results. (a) No.7 coal seam; (b) No.8 coal seam; (c) No.12_-1_ coal seam; (d) No.9 coal seam; and (e) No.5 coal seam.

Fig 15 shows the field measurement results. The rationality of shield selection is verified by analysis of SSC. Moreover, a basis is provided to study the relationship between the shield and roof. Furthermore, the adaptability of the shield is also analyzed.

Table 17 shows the SSC results of field measurement. Based on the RSSC experienced selection, the maximum support load utilization in each coal seam is 68.84%, 82.56%, 73.59%, 76.67%, and 69.95%, respectively. Comprehensively considering the results of SSC in each coal seam, the shield selection is unreasonable excluding the No.8 coal seam, where the maximum support load utilization is less than 80%. That means the RSSC is too high resulting in the shield being unable to achieve the optimal support condition.

**Table 17:** SSC results of field measurement in each coal seam.

Coal seam	Measured ASSP	Measured SSC	RSSC experienced selection	Maximum support load utilization
No.7	22.9~27.4MPa	3682.41 ~ 4406.03kN	6400kN	68.84%
No.8	24.2~27.4MPa	2978.46 ~ 3963.08kN	4800kN	82.56%
No.12_-1_	20.6~28.7MPa	2983.56 ~ 3532.31kN	4800kN	73.59%
No.9	24.0~29.9MPa	2953.80 ~ 3680.00kN	4800kN	76.67%
No.5	13.2~28.4MPa	1625.62 ~ 3497.54kN	5000kN	69.95%

**Fig 16:**
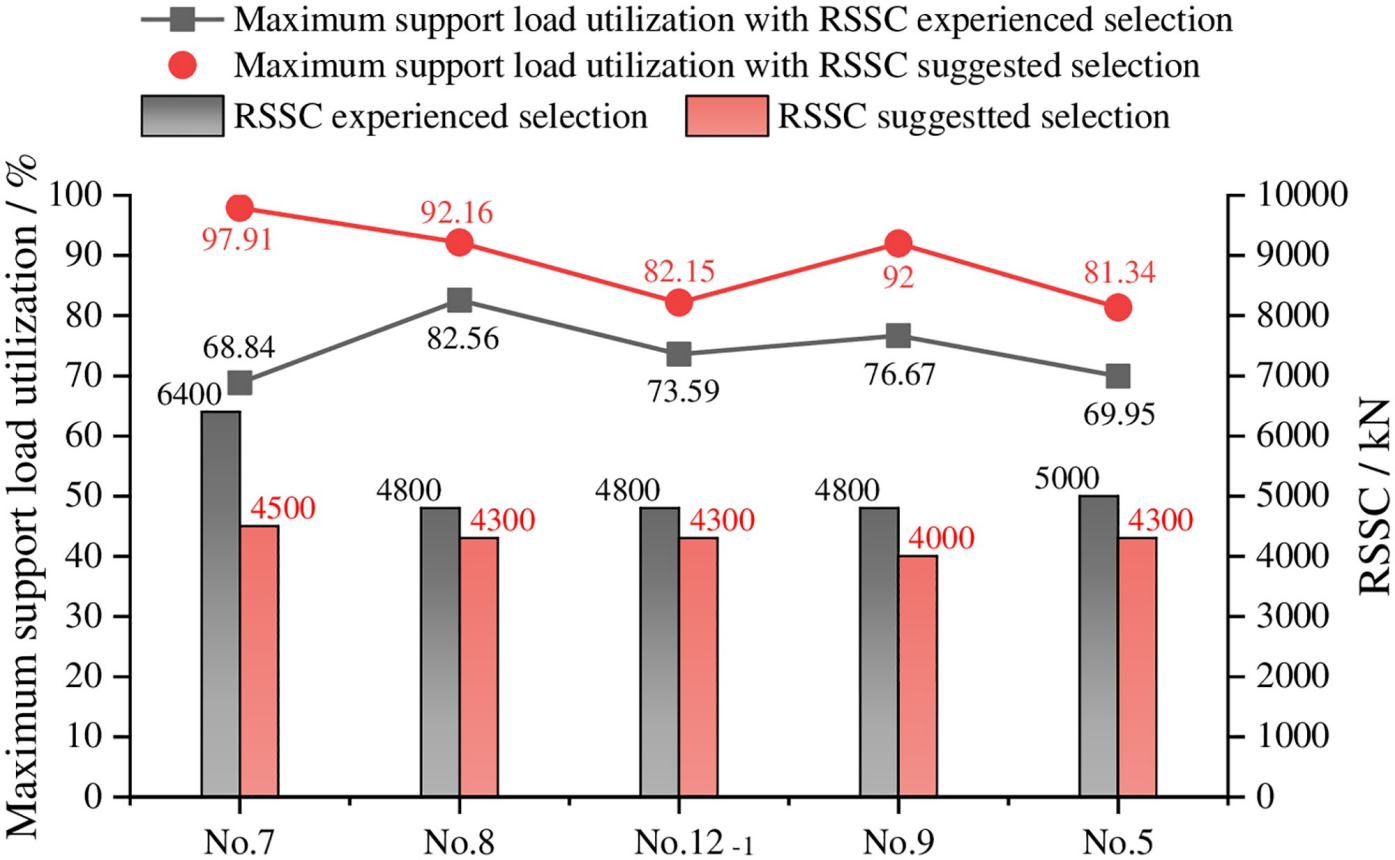
Compared results of the RSSC suggested and experienced selections.

Table 18 shows the SSC results of the calculation. According to the calculation results in *Analysis of SSC in Each Coal Seam*, the RSSC suggested selection is 4500kN, 4300kN, 4300kN, 4000kN, and 4300kN, respectively. Furthermore, the maximum support load utilization in each coal seam is 97.91%, 92.16%, 82.15%, 92% and 81.34%, respectively. Fig 16 shows the compared results of the RSSC experienced and suggested selection. Obviously, the maximum support load utilization increases by 29.07%, 9.6%, 8.57%, 15.33%, and 11.39%, compared with the RSSC experienced selection. The SSC has been fully utilized.

**Table 18:** SSC results of calculation in each coal seam.

Coal seam	Calculated MSSC	RSSC suggested selection	Maximum support load utilization
No.7	3948.55kN	4500kN	97.91%
No.8	4018.32kN	4300kN	92.16%
No.12_-1_	4101.63kN	4300kN	82.15%
No.9	3560.03kN	4000kN	92.00%
No.5	4015.30kN	4300kN	81.34%

## Conclusions

1. According to whether the fractured rock blocks generated by the main roof are articulated, there are two roof structure mechanical models to calculate the MSSC. The first type is the articulated structure of the fractured rock blocks generated by the main roof, and the MSSC per unit width is composed of the load of the immediate roof and the force acting on the shield when the rock blocks of the voussoir beam are generated by thick main roof slip instability. The second type is the unarticulated structure of the fractured rock blocks generated by the main roof, and the MSSC per unit width is also composed of the load of the immediate roof and the force acting on the shield of the rock blocks generated by the thick main roof. According to whether the upper coal seam is mined, the load on the rock blocks A and B can be calculated in two ways, which are a given load of overburden strata and a given load of loose body.
2. A selection step of roof structure calculation method of MSSC is put forward according to whether the upper coal seam has been mined firstly, whether the lower coal seam is influenced by the upper coal seam mining secondly, and whether there is a thick and hard stratum between the upper coal seam and the lower coal seam lastly.
3. Taking the close-multiple coal seam in the Qianjiaying coal mine with the coordinated mining as a case study, there are three calculation models to calculate the MSSC. The roof structure after No.7, No.12_-1,_ and No.5 coal seam mining is a voussoir beam balanced structure. The roof structure after No.8 coal seam mining is a balanced structure with a given load of loose body. And the roof structure after No.9 coal seam mining is a voussoir beam balanced structure with a given load of loose body.
4. As for the voussoir beam balanced structure and voussoir beam balanced structure with a given load of loose body, the MSSC includes the weight of the immediate roof and the force acting on the shield when the rock blocks of the voussoir beam are generated by the thick main roof slip instability. But the load on the rock blocks A and B of the voussoir beam generated by the main roof is different depending on whether the upper coal seam is mined. As for the balanced structure with a given load of loose body, the MSSC includes the weight of the immediate roof and a given load of loose body.
5. Through the detailed calculation of the MSSC of each coal seam mining in the Qianjiaying coal mine, the MSSC of No.7, No.8, No.12_-1_, No.9, and No.5 coal seam is 3948.55 kN, 4018.32 kN, 4101.63 kN, 3560.03 kN, and 4015.30 kN, respectively. So, the suggested RSSC of each coal seam is 4500 kN, 4300 kN, 4300 kN, 4000 kN, and 4300 kN, respectively. By field measurement of the ASSP or SSC, the RSSC experienced selection of each coal seam in the Qianjiaying coal mine is unreasonable with low support load utilization. However, after adopting the RSSC suggested selection in each coal seam, the support load utilization increased by 29.07%, 9.6%, 8.57%, 15.33%, and 11.39%.
